# Bilingualism: A Global Public Health Strategy for Healthy Cognitive Aging

**DOI:** 10.3389/fneur.2021.628368

**Published:** 2021-04-15

**Authors:** Sahan Benedict Mendis, Vanessa Raymont, Naji Tabet

**Affiliations:** ^1^South London and Maudsley NHS Foundation Trust, London, United Kingdom; ^2^Oxford Brain Health Clinical Trials Unit, Oxford, United Kingdom; ^3^Center for Dementia Studies, Brighton and Sussex Medical School, Brighton, United Kingdom

**Keywords:** bilingualism, cognitive reserve, dementia, healthy cognitive aging, global public health, low and middle income countries, contextual challenges

## Abstract

Dementia is a global public health priority which cost global societies $818 billion in 2015 and is disproportionately impacting low and middle-income countries (LMICs). With limited availability of disease modifying drugs to treat Alzheimer's disease (AD), researchers have increasingly focused on preventative strategies which may promote healthy cognitive aging and mitigate the risk of cognitive impairment in aging. Lifelong bilingualism has been presented as both a highly debated and promising cognitive reserve factor which has been associated with better cognitive outcomes in aging. A recent metanalysis has suggested that bilingual individuals present on average 4.05 years later with the clinical features of AD than monolinguals. Bilinguals are also diagnosed with AD ~2.0 years later than monolingual counterparts. In this perspective piece we critically evaluate the findings of this metanalysis and consider the specific implications of these findings to LMICs. Furthermore, we appraise the major epidemiological studies conducted globally on bilingualism and the onset of dementia. We consider how both impactful and robust studies of bilingualism and cognition in older age may be conducted in LMICs. Given the limited expenditure and resources available in LMICs and minimal successes of clinical trials of disease modifying drugs we propose that bilingualism should be positioned as an important and specific public health strategy for maintaining healthy cognitive aging in LMICs. Finally, we reflect upon the scope of implementing bilingualism within the education systems of LMICs and the promotion of bilingualism as a healthy cognitive aging initiative within government policy.

## Introduction

Twenty first century societies are rapidly transitioning to aging populations which are often characterized by a burden of age related conditions such as dementia (1). There are about 50 million individuals living with dementia (2); a figure that is forecasted to increase to 115 million by the year 2050 (3). The global economic cost of dementia was measured at US $818 billion in 2015 (4). The burden of dementia significantly impacts low and middle countries (LMICs)^1^ and by 2050 we anticipate that 71% of all people living with dementia will reside in LMICs (1).

Whilst there have been limited successes of clinical trials and disease modifying drugs (6), researchers have focused on developing public health strategies that may promote healthy aging and support the delay of onset of dementia (7). This approach may be germane to LMICs where minimal resources and unstable health systems may make running of clinical trials more challenging and logistically difficult. In these settings, dementia may be viewed as part of a normal aging process and a highly stigmatized condition and associated with limited provision of care for individuals with dementia. These additional factors may complicate how dementia prevention is addressed in non-communicable disease (NCD) policies in LMIC settings (8). There are potential promising economic benefits of delaying the onset of dementia. A recent study has demonstrated that a 1 year delay of onset of dementia reduces formal costs in 2030 by $70 billion and informal costs by $43 billion compared to no delay on dementia onset (9).

Cognitive reserve is a hypothetical construct which posits that enriching lifetime experiences and activities support the brain in mitigating the impact of pathological damage over time (10–15). This may enable individuals to cope better with brain damage and sustain greater degrees of brain damage before demonstrating functional deficits (10). Cognitive reserve factors have been associated with the delay of onset of dementia and better cognitive outcomes in aging (16). These factors include educational attainment (17), the cohesion of social networks (18), occupational complexity (19), enhanced physical activity, and cognitively stimulating activities (20). Bilingualism has been positioned as a powerful cognitive reserve factor (21) which may be associated with the delay in onset of dementia. Encouragingly cognitive reserve may be malleable even in older age which may provide significant opportunities for interventional studies of cognition in later life (22).

A recent metanalysis by Paulavicius et al. (23) revealed that studies that explored the relationship between bilingualism and age of onset of dementia revealed an average of 4.5 years in the delay of presentation of dementia amongst bilinguals. In this perspective piece, we critically review bilingualism as a cognitive reserve factor and examine the key studies of bilingualism explored in both high income countries (HICs) and LMICs. We discuss the implications of these findings to a global health context. We commentate on the key study findings of the above metanalysis. We address some of methodological limitations of the current evidence and suggest ways in which these can be overcome.

We propose that incorporating bilingualism into dementia public health policy to delay the onset of dementia is an important and specific strategy in maintaining healthy cognitive aging in LMICs. We reflect how bilingualism can be incorporated into governmental and educational policy and overall health strategy in LMICs settings and the challenges associated with this.

## What is Bilingualism?

Bilingualism can be classified as individuals' ability to communicate using two languages either actively using speech or listening, or passively using writing, reading, or listening. The bilingual experience is a dynamic process and proficiency may differ according to the level of exposure to other users of each language and level of opportunity to use each language (24). Bilinguals can be described as either simultaneous; where an individual is exposed to both languages to a significant degree from birth, or alternatively sequential, where meaningful exposure to the second language is developed after the age of three (24). Bilingualism does not require any special education or intellectual ability. It is a common phenomenon, and ~50 % of the global population are proposed to have some bilingual or plurilingualism proficiency (25). Bilingual children and adults may experience difficulties with “lexical access” and reduced verbal fluency and this may lead to “tip of the tongue” experiences (26). Bilingual proficiency can be observed in different social and cultural contexts. Daily contact with two languages is observed globally in diverse settings, such as Europe (Switzerland, Belgium, and Luxemburg), Asia (India, Philippines), Africa (Senegal, South Africa), and North America (Canada).

## The Case for Bilingualism as a Cognitive Reserve Factor

Lifelong bilingualism has been positioned as a cognitive reserve factor (21) and promoting buffering against age related cognitive decline. There are two distinct models of cognitive reserve; brain reserve and neuronal compensation (10, 27). The brain reserve model asserts that existing brain networks are more resilient toward deregulation because of heightened efficiency. This may be mediated through enhanced “brain hardware” (10), which may be practically achieved through increased dendritic volume, brain synapses or overall brain volume (10). These networks may facilitate brain activity when performing more complex tasks and may enable the brain to cope more effectively with degeneration. In neuronal compensation, the brain recruits additional areas that are not normally used to perform the skills that have been lost in the degenerated brain (10). Other models of cognitive reserve include a life course perspective (14), scaffolding theory (28), or the concept of brain maintenance (15).

## Neuroimaging

Bilinguals simultaneously attend to two competing languages for selection which may induce neuroplasticity (29) and lead to remodeling of brain architecture and function (30). Schweizer and colleagues (21) who analyzed linear CT brain looking at brain atrophy, discerned that greater amounts of neuropathology are needed before the clinical symptoms of AD become apparent in bilinguals. Similarly, another study using PET demonstrated that bilinguals had greater regional glucose uptake than monolinguals (31). Bilingual brains have been shown to have specific activity in the frontotemporal and subcortical networks which are involved in interference inhibition, and may facilitate language switching (32). This was not demonstrated in monolinguals. Bilinguals may have increased capacity for conflict resolution through augmentation of anterior cingulate cortex activity (33).

The bilingual experience may promote more widely distributed neural activity (30), recruitment of overlapping neural regions which are not usually found in monolinguals (34) or enhancing neural activity in regions involved in executive function (30). Bilingual adults may display greater gray matter volume particular in the anterior cingulate cortex (35) parietal lobes (36), corpus callosum (37) the basal ganglia (30, 38) and the frontoparietal network (FPN) (30). Bilinguals show greater white matter integrity and gray matter functional connectivity compared to monolinguals (30). Functional MRI studies reveal that although bilinguals have equal performance in non-verbal executive tasks there is less frontal activation than monolinguals (30). This suggests that bilinguals do not rely on “top down” mechanisms in cognitive functions (30). Overall, researchers suggest that the shift from anterior to posterior brain activation amongst bilinguals “anterior-to-posterior and subcortical shift” /BAPSS (30) may provide some evidence why bilingualism is associated with improved cognitive performance in older age and delayed onset of dementia.

## Neuropsychology, Epidemiology, and Lab Studies

The positive findings which are reflected in the neuroimaging studies of bilinguals are also depicted in multiple neuropsychological and epidemiological studies of bilingual adults. Bilinguals have been shown to outperform monolinguals in tests of executive function, such as cognitive control (39), working memory (40), inhibition (41), and attention (42). However, other researchers may refute findings linking bilingualism and improved executive function because many studies may be limited by small sample sizes (43), socioeconomic factors (44), education, and geographical location (45). These factors are known to have significant impact on performance of executive function (46). By contrast, Nichols et al. (47) compared the performance 11,041 (5,994 monolinguals and 5,047 bilinguals) participants on a battery of 12 executive tasks and found there was no significant difference between the two groups on executive function. These findings were independent of case mix factors (47). However, it is important to note that this study only included 744 people in the matched bilingual and monolingual sample and defined bilingualism based on a single question “How many languages do you speak (47)?” This simplistic and imprecise approach to measuring bilingual proficiency may misrepresent the nuanced complexities of bilingual proficiency and we suggest the findings of this study should be interpreted with some caution. The overall findings suggest that bilingualism and executive function research should be conducted in diverse sociocultural milieus to ascertain whether the bilingual advantage applies in different contexts.

A 12 year longitudinal Israel based study of 814 elderly Jewish people revealed trilinguals performed better on cognitive tasks than monolinguals and bilinguals (48). These findings were independent of educational achievement, occupation, age, place of birth, and immigration (48). A study explored 853 participants who were recruited into the Lothian cohort 1936 study (49). This followed adults whose age 11 IQ was measured as part of the Scottish Mental Survey 1947 (49). Repeated cognitive testing between 2008 and 2010 revealed that bilingual participants performed better than monolinguals in both reading and executive function tests, as well as in tests of intelligence (49). Another study observed that bilinguals with amnesic-type mild cognitive impairment had a reduced rate of conversion to AD compared to monolingual counterparts (50). This delay was not demonstrated in mild cognitive impairment participants with multiple domain deficits (50). Bilinguals are twice more likely to recover cognitively from stroke than monolinguals (51). Bilingualism has been associated with better ratio of CSF AD biomarkers (52).

## Does Bilingualism Delay the Onset of Dementia? Key Findings From a Systematic Review

Having explored the contextual evidence supporting bilingualism as a cognitive reserve factor, we now evaluate the systematic review from Paulavicius et al. (23) exploring bilingualism and age of onset of dementia and the specific epidemiological studies exploring this relationship.

This systematic review reported findings from eight studies which examined the relationship between bilingualism and the age of onset of dementia. Metanalysis from these studies determined that bilinguals (53) with AD presented with delayed clinical features (694 individuals; mean difference MD 4.05 years; 95% CI:1.87–6.22) and are diagnosed (1,012 participants: MD 2.0 years; 95% CI 0.08–3.92) (23). This study incorporated studies which were cross sectional, cohort, case control or retrospective in design. Six of the selected studies consisted of only AD patients and four of the studies had a mixture of immigrant and non-immigrant populations. The study pooled data from four studies that had investigated the age of onset of AD symptoms (23). Secondly, five studies which determined the age of AD diagnosis were pooled. All the selected studies were retrospective in design (23). Another systematic review which examined the impact of bilingualism on the risk of cognitive decline found that bilingualism was not associated with a reduced incidence of dementia (54). This study only included prospective studies and studies of different types of dementia (54). Overall, studies suggest that bilingualism is associated with a delayed onset of clinical presentation of dementia but not reduced risk of developing dementia or reduced incidence of dementia (54–56).

## Studies of Bilingualism in HICs

Tables 1, 2, respectively outlines the key studies of bilingualism conducted in HICs and LMICs. Twelve key studies of bilingualism were conducted in HICs (53, 57–59, 61, 63–66, 72, 73) and all investigated spoken bilingualism. Of the 12 studies, six studies were conducted in USA (58, 61–64, 67), four studies in Canada (53, 57, 59, 65), one study in Belgium (60) and one study in Wales (66). Nine studies involved a retrospective analysis of bilinguals vs. monolinguals. Eight studies revealed a delay of onset of dementia in bilinguals whilst four studies did not find a difference between monolinguals and bilinguals (63–66). Three studies were prospective and had a cohort or cross-sectional design (63–65) and did not find a delay of onset of dementia associated with bilingualism. All but one Canadian study revealed a positive relationship between bilingualism and delayed onset of dementia (53, 57, 59). Similar findings were found in the Belgium study (60). The sample size for these studies ranged from 86 to 1,616 subjects. Gollan et al. (58) explored bilingual objectively measures of linguistic proficiency using the Boston Naming Task. Zahodne et al. (63) also used an objective measure of English reading level. All studies used different operational definitions of bilingualism and different linguistic profiles and varying pairs of languages.

**Table 1:** Studies of bilingualism and age of onset of dementia in HICS.

**Name, country of study participants and year**	**Description of study design**	**Key study findings**	**Methodological limitations**	**Conclusions**	**Additional commentary covariables**
Bialystok et al. (53) Canada	This study examined whether bilingualism was associated with delay of onset of dementia. Retrospective analysis of 184 patients attending Baycrest in Toronto memory clinic 93 were bilinguals and 91 were monolinguals with dementia. Onset of cognitive impairment reports, and age of diagnosis of cognitive symptoms noted. Bilinguals defined as those who spent the majority of their lives, at least from early adulthood, regularly using at least two languages.	The difference between monolinguals and bilinguals of 4.1 years in age of onset of symptoms f_(1, 178)_ = 9.16, *p* < 0.003, with no difference between men and women, F < 1. The power of this effect with α = 0.05 is 0.87 Bilinguals were 3.2 years older than monolinguals at the time of the initial clinic appointment, a difference that was also significant, F_(1, 180)_ = 5.93, *p* < 0.02	Subject to recall bias. 38 patients unaccounted. Bilingual participants were mainly francophone and immigrants 81/93.	Bilingualism may delay the age of onset of clinical features of dementia.	Immigration may propagate healthy worker effect in the bilingual population. Retrospective sample Relatively small bilingual population. Study controlled for gender, occupation and level of education. The bilinguals included speakers of 25 different languages
Craik et al. (57) Canada	211 consecutive patients attending clinic in Toronto with AD. 102 bilinguals and 109 monolinguals were selected. Age of onset of cognitive impairment and demographic information, such as factors including occupation, education, and linguistic history taken out of 102 bilingual participants and 109 monolingual participants tested. Bilinguals defined as individuals having spent the majority of life, at least from early adulthood, regularly using at least two languages	Bilinguals were diagnosed 4.1 years later than monolinguals F_1, 207_ = 12.02, *p* < 0.0006, and the report of onset of symptoms was 5.3 years later than monolinguals. F_1, 205_ = 16.25, *p* < 0.0001	Majority of bilingual participants were immigrants. 21 different first languages were spoken amongst b Yiddish (*n* = 24), Polish (*n* = 12), Italian (*n* = 11), Hungarian (*n* = 9), and French (*n* = 7). Questionnaires about fluency of languages given to participants but was not formally assessed.	Bilingualism delays the onset cognitive symptoms.	No effect from immigration, and monolinguals achieved more formal education. Groups were very similar on occupational and cognitive attainment. Immigration status was analyzed as an independent factor.
Gollan et al. (58) USA	This study examined the impact of increasing bilingual proficiency in Spanish speaking AD bilingual patients in terms of age of onset of diagnosis. Bilingualism proficiency and age of diagnosis of Alzheimer's disease and age of diagnosis assessed in 44 participants. Spanish and English-speaking bilinguals. Degree of bilingualism was measured using the Boston naming test, and bilingual index. These were participants attending the UCSD Alzheimer's research Center.	Later age of diagnosis on more bilingually proficient participants. Greatest difference found in those with low levels of education and those with Spanish dominant linguistic proficiency Being more bilingually proficient may delay the onset of cognitive symptoms of dementia.	Only bilinguals studied in this project. Retrospective analysis	There may be an upper limit to the level of protection conveyed by bilinguals, as greatest delay in cognitive symptoms observed was most pronounced in the least educated participants.	Objective measures of bilingual proficiency using Boston naming test.
Bialystok et al. (59) Canada	Study investigating the relationship between bilingualism and age of onset of cognitive symptoms of dementia and rate of deterioration of cognitive symptoms in monolinguals and bilinguals with dementia. Participants were selected from the Sam and Ida Ross Memory Clinic at Baycrest, Toronto, Canada. 74 patients with MCI and 75 patients with AD (35 monolinguals) and (40 bilingual) and participants were followed up over a year. All patients were interviewed to obtain details of their language use, onset of their condition, and lifestyle habits. Bilinguals were defined as those who had spent most of their lives beginning at least in early adulthood, speaking two or more languages fluently on a daily or at least weekly basis.	Significant delay in the onset of cognitive symptoms in patients with MCI and AD (3.2 and 7.2 years, respectively). Mean age of onset of dementia monolinguals was 70.9 vs. 78.2 in bilinguals. The rate of executive function decline was approximately the same in both bilinguals and monolinguals with Alzheimer's disease. Bilinguals were older than monolinguals for both onset of symptoms [F_(1, 145)_ = 10.75, *p* =.001] and age of first clinic visit [F_(1, 146)_ = 9.35, *p* =.003].	Prospective assessment of the rate of decline of symptoms in bilingual and monolingual groups. Retrospective analysis of the date of diagnosis of dementia. All subjects were proficient in English, but bilinguals additionally spoke other languages such as Farsi, French, Italian, Russian, and Yiddish.	Bilingualism delays the age of onset of AD	47% of the patients from the Craik et al. (57) study was also used in this study. The delayed onset of cognitive symptoms in the bilingual group were independent of lifestyle factors. Language and Social Background Questionnaire (LSBQ) assessed immigration history, education, and language use. Onset of symptoms interview explored when next of kin noticed the symptoms of dementia.
Woumans et al. (60) Belgium	The study aim was to evaluate the age of onset of dementia in monolinguals or bilinguals in a sample in Belgium. 69 monolinguals with AD and 65 bilinguals with AD were identified from 2 university hospitals in Ghent and Brussels. Non-immigrant sample of bilingual participants were recruited. Participants were considered bilingual if they rated themselves as “good” or higher for all four L2 skills and spoke this L2 at least weekly before and now were obtained from patient and caregiver interviews. Multiple linear regression performed.	A delay of 4.6 years in clinical manifestation and 4.8 years in diagnosis of dementia in bilinguals compared to monolinguals. Group [F_(1, 109)_ = 6.18, *p* =.014, Beta = 4.64 years], Average age of manifestation of dementia in monolinguals was 71.5 and bilinguals was 76.1	Retrospective study	Bilingualism delays the clinical manifestation of dementia.	Age of language 2 acquisition did not affect the findings. Bilinguals consisted of a combination of French and Dutch. Linguistic history and social background information Proficiency measured by Likert scale and frequency of use of language assessed. No objective measurement of bilingual proficiency.
Mendez et al. (61) USA	The study aim was to evaluate the effects of bilingualism on the age of diagnosis of dementia. In clinics in California USA with a large immigration population 253 patients with probable early onset AD identified and investigated for demographic variables, native language nature of presentation, ages of onset and presentation. Mini-Mental State Examination, digital	74 bilinguals (29%) and 179 monolinguals were recruited in the study. There was a variety of L1s Bilinguals had significant delays in age of onset of dementia (*p* = 0003) and age of presentation (*t* = −3.03; df 251, *p* = 0.003) Bilinguals had worse MMSE scores on presentation.	Retrospective study design Logistic regression performed for bilingual and monolingual groups.	Bilingualism delays the onset of dementia	Most of the bilinguals were from immigrant population who spoke a variety of L1s (Farsi, Spanish, Chinese, Tagalog, Arabic etc) Majority of bilinguals regressed back to their native L1. Amongst bilinguals language use in the first years of life, the later acquisition of English, immigrant status, the proficiency in using both
	spans, word fluencies, naming, and memory were measured.				languages on a daily basis, and change in language used.
de Leon et al. (62) USA	This retrospective study explored the difference in age of onset of dementia in bilinguals and monolinguals in 287 well-characterized participants with either amnestic Alzheimer's dementia or logopenic variant primary progressive aphasia (lvPPA) Individuals were selected from those seen at the University of California, San Francisco Memory and Aging Center (MAC)	Of the 287 participants, 247 were monolinguals and 40 participants were monolingual. Of the 246 monolinguals 179 had amnesic AD and 63 monolinguals had IvPPA. Amongst the bilinguals 28 had Amnesic AD and 16 had IvPPA. Participants who spoke two or more languages were classed as bilinguals. If charts did not state information regarding exposure to or experience with a second language, they are monolingual. lvPPA cohort, bilingual speakers were significantly older than monolinguals at the time of diagnosis Bilinguals(M = 68.2) Monolingual(M = 62.8) for the monolingual This finding was not found in Amnesic AD	Retrospective design No objective rating of bilingual proficiency. Bilinguals were more likely to be immigrants to USA.	Bilingualism was associated with a significant delay in onset of dementia in IvPPA patients. This difference was not observed in Amnesic AD.	Study excluded participants who enrolled in second language classes for only a few years without ongoing experience. Study excluded individuals that had immigrated to a country which have a majority different primary language but it was not evident whether they were in formal school or employed in their adopted country or participants expressed minimal proficiency in a second language. Two raters independently determined monolingual or bilingual status for each patient. Analyses of covariance (ANCOVAs) were used to assess the effects of bilingualism and clinical diagnosis on age at symptom onset.
Zahodne et al. (63) USA	Large prospective USA study investigating the Spanish speaking community of initially non-demented individuals living in Manhattan. 1,067 participants from the Washington/Hamilton Heights Inwood Columbia Aging Project (WHICAP) who were tested in Spanish and followed at 18–24 month intervals for up to 23 years.	282 of the participants converted to dementia. Bilingualism was not associated with a reduced conversion or reduced rates of cognitive decline. Bilingualism was associated with better performance in memory tasks and executive function.	No objective measure of Spanish proficiency taken.	Bilingualism may not be associated with delayed impairment of cognitive function.	Bilingualism was tested by self-rating and objective test in reading ability in English was conducted.
Lawton et al. 2014 (64) USA	Secondary analysis of 81 (55 Alzheimer's Disease 26 Vascular Dementia) participants who developed dementia. Study sample taken from the Sacramento Area Latino Study on Aging cohort study. 1,789 Hispanic Americans were enrolled for this study and the participants were self-identified Hispanics and none of the participants had dementia at the start of the study. These 81 community dwelling participants performed cognitive tests, and the age of diagnosis were determined.	Mean age of diagnosis was 81.1 in monolingual group and 79.9 in bilingual group. ANOVA revealed that the mean age of dementia diagnosis of the bilingual participants (79.31 years) was not significantly different from that of the monolingual participants (81.10), F_(1, 77)_ = 1.27, *p* = 0.26, η2p = 0.02.	Bilingualism not associated with delay of onset of dementia.	Over 50% of the population were immigrants to USA. 57% of the bilingual group were multilingual and were not analyzed separately to bilingual group. Small study sample. Language proficiency in each language not objectively measured. Small study sample	Large study sample taken Hispanic bilinguals only targeted. Bilinguals were significantly better educated than monolinguals with dementia. No significant difference in education levels in US born bilinguals or monolinguals. Likert scales used to identify frequency of language use.
	Cognitive tests performed included the MMSE English Neuropsychological Assessment Scale.				
Yeung et al. (65) Canada	Study explored whether bilingualism is associated with dementia in cross sectional or prospective analyses of older adults. 1,616 community living older adults were assessed and followed 5 years later. Measures included subjective memory loss, modified MMSE Dementia defined as cut off on modified MMSE. Language status defined as first language English, bilingual English, English as second language.	No association between speaking more than one language and dementia. English as a second language participants had poorer education, and more likely to be diagnosed with dementia compared to those speaking English as a first language.	Bilingualism is not associated with a delayed onset of dementia.	Original sample had 2,890. Over 1,200 participants lost to follow up, 443 refused, 131 participants had missing data, Self-reported measurement of education and multilingualism were documented. No independent measure of bilingualism. 9.6% cognitive impairment in English as second language group. Differences in participant numbers between English as first language and English as second language group. Modified MMSE poor indicator of cognitive dysfunction No neuroimaging information provided. 3MS- is highly English specific and therefore ESL group may find it difficult to perform.	Overall poorly designed study and big losses to follow up Self-reporting of language proficiency leading to bias. Large losses to follow up. Different sample sizes in different groups. Community based study. Large disparity in the levels of education between the groups which may have resulted in bias. Genetic factors not measured. Measures of cognitive ability were poor. Study did not have specific age of onset of dementia information.
Clare et al. (66) Wales	Welsh cross sectional cohort study compared the time of diagnosis of Alzheimer's disease in 49 monolingual English speakers and bilingual 37 and English and Welsh speakers. These participants were then requested to perform executive function and neuropsychological testing.	Bilinguals did not show significant advantages in executive function compared to monolinguals, but there was some increased ability in inhibition and conflict resolution in bilinguals.	A non-significant delay in cognitive impairment diagnosed in bilinguals with dementia compared to monolinguals.	Only 24 of 37 bilingual Welsh and English participants were selected for performing executive function cognitive tasks.	Bilinguals came in touch of medical care later than monolinguals with dementia. Bilinguals shared a common societal and cultural milieu. Bilinguals were found to be significantly less educated, and more likely to be on cholinesterase inhibitors.
	Language questionnaire was created to explore the level of language proficiency. Cognitive reserve info ascertained by lifetime of experience of questionnaire. Variety of executive function tests given to participants. All participants had a screening MMSE score of 18/30. Participants selected from the neurodem research register. Power calculations revealed that there needed to be 42 participants in both groups in order to show a significant statistical different in the age of the onset of dementia. Structured interview was given in the language of choice.	Bilinguals were diagnosed not significantly 3 years later than monolinguals, but were also more significantly cognitively impaired than monolinguals.		Underpowered study particularly with small bilingual population may have contributed toward the inconclusive results.	Participants were assessed 1.5–2 years post-diagnosis. Higher dropout rate in bilinguals with Alzheimer's compared to monolinguals. Difficult to recruit bilinguals to the study.
Akhlaghipour et al. (67) USA	This retrospective study examined the relationship between speaking more than one language and the age of onset of the clinical symptoms of Alzheimer's disease, and (2.) to investigate if there is asymmetrical language impairment with reversion to L1(dominant language) once there is clinical dementia. This identified 74 bilingual and 179 monolingual patients. Dependent variables were age of onset and presentation.	Bilingualism was associated with statistically significant delay in ages of onset and presentation of clinical dementia (*p* = 0.003). MMSE score was significantly lower in monolingual compared to bilinguals (*p* = 0.004). Improved scores in F word fluency, category fluency, and delayed verbal recall among bilinguals compared to monolingual patients.	Bilingualism is associated with delayed onset of dementia	Only supplementary information provided No information provided on the proficiency of languages or assessment of proficiency of languages.	No documented measurement of the average number of years of delay of onset of dementia in bilinguals compared to monolinguals.

**Table 2:** Studies of Bilingualism and age of onset of dementia in LMICs.

**Name, country of study participants and year**	**Description of study design**	**Key study findings**	**Methodological limitations**	**Conclusions**	**Additional commentary covariables**
Alladi et al. (68) India	Case records of 648 patients with dementia (391 bilingual) diagnosed at a specialized memory clinic in Hyderabad India were appraised. The subjects had AD, *n* = 240FTD, *n* = 116 vascular dementia *N*= 189 Lewy Body Dementia *n*= 55 Mixed dementia *N* = 48	Univariate GLM analysis showed that bilingualism was significantly associated with delay of dementia [F_(1, 458)_ = 4.89, *p* = 0.027) This finding was independent of casemix factors Bilinguals with dementia presented on average 4.5 years later than the monolinguals. 3.2 year delay in bilinguals with AD 3.7 year delay in bilinguals with Vascular dementia 6 year delay in bilinguals with Frontotemporal dementia. Amongst illiterate bilinguals delay of onset of dementia was 6 years (65.0 vs. 59.0 years, *p* = 0.03) These findings were independent of confounding variables No additional benefit in speaking more than two languages	Retrospective analysis of case records Spoken fluency in languages not formally assessed	Bilingualism may delay the onset of cognitive symptoms associated with dementia independent of other risk factors. Protection was also found in illiterate bilinguals; therefore, results may be independent of the level of educational attainment.	Diverse linguistic groups in study sample including speakers of Telugu-, Dakkhini-, and the Hindi Case mix factors measured literacy, years of education, sex, dementia subtype, vascular risk factors, stroke, occupational status, rural/urban dwelling, family history of dementia, and dementia severity
Alladi et al. (69) India	This study examines whether bilingualism delays the age of onset of frontotemporal dementia FTD. Dementia patients were split into aphasic and behavioral groups. Case recordings of 193 patients presenting with FTD of which 121 were bilingual and age of onset of first symptoms were compared between bilinguals and monolinguals. Participants were selected from those attending dementia clinics in Hyderabad	The age of dementia in bilingual behavioral FTD (62.6) was over 6 years delayed than monolinguals (56.6, *p* = 0.006). No difference was found in aphasic groups. This delay was independent of rural/urban dwelling, literacy, and education, gender and family history of dementia.	Retrospective design Monolingual and bilinguals were compared using independent samples *t*-tests. One-way test of variance.	Bilingualism delays the onset of dementia in only behavioral variants and not aphasic groups.	A variety of different dementias including behavioral variant of FTD, semantic dementia, corticobasal dementia, progressive supranuclear palsy, and FTD-motor neuron disease. The languages combinations included Telugu and Hindi, Telegu, English and Hindi and Telugu and Dakkani. Spoken fluency was not formally assessed.
Ellajosyula et al. (70) India	Case records of patients diagnosed with dementia in a South Indian clinic were selected. There were 183 patients diagnosed with dementia 109 AD and 74 FTD. 55 30.1% were monolinguals and 128 69.9 % bilinguals or multilinguals. Age of onset of dementia ascertained.	No significant difference between bilinguals/multilinguals and the age of onset of dementia.	Bilingualism may not delay the onset of cognitive symptoms of dementia	Relatively small monolingual population. Retrospective analysis	Bilinguals and multilinguals were analyzed together. Case records were analyzed
Zheng Y et al. (71) China	Retrospective study exploring whether Cantonese /Mandarin bilingualism is associated with a delayed onset of dementia. 29 patients diagnosed with probable AD, including 48 Cantonese monolinguals, 20 Mandarin monolinguals, and 61 Cantonese/Mandarin bilinguals were analyzed.	Cantonese/Mandarin bilinguals were found to be an older age at AD onset, and were 5.5 years older at the first clinic visit than Mandarin monolinguals and Cantonese monolinguals Multiple linear regression analysis performed on study participants which revealed that bilingualism was statistically significantly associated with dementia delay. (*P* = 5.497, *p* = 0.017)	Bilingualism associated with delayed onset of dementia	Small study sample Retrospective analysis	

## Studies of Bilingualism and Age of Onset of Dementia in LMICs

Alladi and colleagues (68) evaluated hospital records of 648 patients of which 391 were bilinguals diagnosed with dementia in specialist clinics in Hyderabad, India and retrospectively evaluated age of diagnosis. This study examined patients with a variety of dementia diagnosis' including vascular dementia, Alzheimer's Disease (AD) and frontotemporal dementia (FTD) (68). This study identified that the bilinguals' mean age of dementia onset was 4.5 years later than monolinguals (68). Bilingualism was significantly associated with the delay of age of onset of dementia, with generalized linear modeling analysis revealing a significant level [*F*_(1, 458)_ = 4.89, *p* = 0.027] after adjustment for immigration, socioeconomic status, illiteracy, education, and residence in rural and urban areas, number of languages spoken and occupational status (68). The study participants were from an autochthonous population where both the monolingual and bilingual participants were born and raised in India (68). This study evaluated important covariates as described above and determined that the findings were independent of these factors (68). In illiterate bilinguals the delay of onset of dementia was 6 years compared to monolingual counterparts (68).

A further Hyderabad based study explored the case records of 193 patients diagnosed with FTD of which 121 were bilingual (69). In this study the age of diagnosis was measured between bilinguals and monolinguals and determined that amongst bilinguals with behavioral variant FTD the age of onset of dementia was 5.7 years later in bilinguals 62.6 vs. 56.5 *p* = 0.006 in monolinguals (69). This finding was independent of the similar case mix factors as observed in the 2007 Hyderabad study (69). Ellajosyula et al. (70) investigated a retrospective South Indian sample of individuals diagnosed with either AD or FTD in a memory clinic. There were 183 patients with dementia where 55 were monolinguals and 129 were bilinguals or multilinguals (70). The study did not find a significant difference in the age of onset of dementia between the two groups (70).

A study explored the relationship between Mandarin and Cantonese bilingualism and age of onset of dementia in 129 patients diagnosed with probable AD, including 48 Cantonese monolinguals, 20 Mandarin monolinguals, and 61 Cantonese/Mandarin bilinguals (71). The study determined that bilingualism was independently associated with delay of onset of dementia [*P* = 5.497, *p* = 0.017 (71)].This study utilized the Bilingual Aphasia Test (BAT) (74) to obtain a detailed language history. All the key studies examined spoken bilingualism only.

## Bilingualism and Cognitive Reserve Research: the Global Context

Our review of key studies investigating bilingualism and the age of the onset of dementia reveal a dearth of studies conducted in LMICs. It may be particularly challenging to directly extrapolate the findings from studies conducted in HICs to LMIC settings (75). Immigration and the potential healthy migrant effect may confound the findings of some studies conducted in HICs. In studies conducted in LMICs bilinguals observed may be from autochthonous populations and in populations where there is a lot of language switching (68). Many contextual challenges including the high prevalence of illiteracy and HIV, unemployment and key differences in employment in both rural and urban settings exist (75). Examples include the unskilled, illiterate craft maker, or illiterate factory worker. The differing ethnic and genetic profiles, such as ApoE may interact or modify the benefits of bilingualism on individuals (75). Other important issues include the high prevalence of head injuries and vascular risk factors and poorly resourced health systems may further complicate assessment and interpretation of research findings (75).

**Figure 1:**
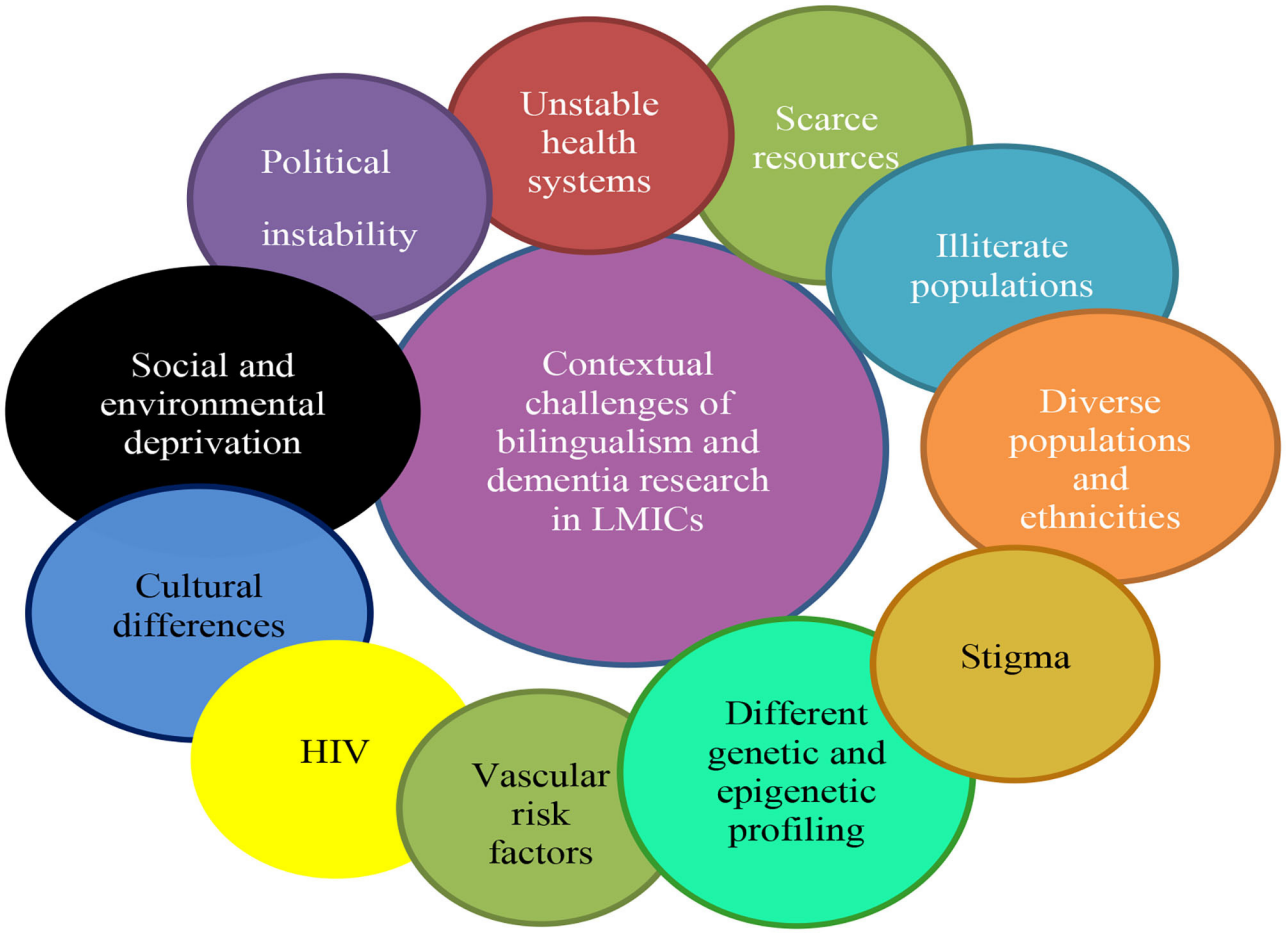
The key contextual challenges of bilingualism and cognitive reserve research in LMICs.

We determine that interactive factors, such as ethnicity, poverty, epigenetics, polluted environments, social deprivation, differing cultures, economics, and politics may have a significant impact in how bilingualism and cognitive reserve research is conducted and interpreted (75). A detailed list of potential interactive factors is outlined in Figure 1. We suggest that tools which formally assess bilingual fluency, such as a culturally amended Boston naming task or BAT, should accompany self-reported fluency of language use. We recommend strict and standardized study definitions of bilingualism should be employed in studies.

Specific challenges may arise when utilizing neuropsychological tests in many LMIC settings for bilingualism and dementia research. Traditionally these tests have been derived for educated and English-speaking western populations and may have limited applicability to other cultures (76, 77). Although, Alladi and colleagues (68) successfully used culturally and linguistically amended versions of the Addenbrookes Cognitive Examination and Dementia Rating Scale there are other specific challenges to consider. High rates of illiteracy in LMIC settings may further complicate the adaptation of these tests (77). There have been attempts to derive culturally unbiased and educationally fair testing (77). Researchers assert that focusing on cognitive tools that emphasizes visual skills, such as the Oxford Cognitive Screen (OCS-Plus) (78) may help to overcome this difficulty. The OCS-Plus is a visual orientated cognitive tool which assesses nine domains of cognition (78). A validation study of the OCS-Plus in a South African study sample in which 45% of the sample did not have any formal education revealed that the OCS-Plus had excellent construct and external validity in detecting cognitive impairment (79). Perhaps we should employ tools, such as OCS Plus in measuring cognition in certain LMICs where low education or literacy levels prevail.

Although the limited neuroimaging resources particularly in rural areas make it challenging to research bilingualism and cognitive reserve, there are examples of big, funded neuroimaging studies conducted in LMICs such as the Health and Aging in Africa: A Longitudinal Study of an INDEPTH Community in South Africa(HAALSI) (79) and the Bangladesh Early Adversity Neuroimaging study (BEAN) (80).We advocate that major global stakeholding funders encourage applicants to present novel approaches, such as researching cognitive reserve in the bilingual brain within LMIC settings. We recommend that both prospective and retrospective studies to be conducted in diverse linguistic and cultural milieu such as South Africa, parts of Latin America and central Asia. Reproducibility of findings in different settings are imperative to understanding how bilingualism and cognitive reserve research is operationalized in a variety of environs.

## Future Research Directions

Finally, we address how future studies of bilingualism and cognitive reserve could be conducted to help us understand the potential benefits of bilingualism in a globalized context. We propose that studies of bilingualism could be performed in high risk and vulnerable populations, such as individuals with mild cognitive impairment (MCI), those with a strong family history of cognitive impairment or genetically susceptible populations. Furthermore, we suggest that prospective studies could be explored in bilingual and monolingual cohorts with a strong vascular history (75), culturally diverse illiterate populations (75), those with limited educational attainment (75), and in specific cultural groups (81). This may help to determine if and how bilingualism may moderate or delay the clinical presentation of cognitive impairment in those with pre-existing risk factors. There may be scope to conduct large longitudinal studies of bilingualism and cognitive aging in densely populated communities in Latin America (82) and mainland China (83) where a range of diverse risk factors are frequently present. Novel and region specific strategies which include the Latin American and Caribbean Consortium on Dementia (LAC-CD1) (84), an approach funded by the Alzheimer's Association and the Global Brain Health Institute may promote the practical implementation of these approaches.

With the advent of neuroimaging modalities and possible increased availability of investigations in LMICs it may be possible to examine how bilingualism may be linked with specific structural neuroimaging findings such as volumetric temporal lobe changes. More relevant information may also be gained from functional magnetic resonance imaging and diffusion tensor imaging. The use of specific neuroimaging techniques, such as fluorodeoxyglucose positron emission tomography (FDG-PET) may be helpful (85), as is the visualization of early amyloid and tau aggregates also assessed through PET. We suggest that studies can also explore the relationship between bilingual proficiency in older adults with the presence of CSF or plasma biomarkers for AD in addition to APOE status.

We emphasize that taking a detailed linguistic history is particularly salient in establishing bilingual proficiency in studies of bilingualism and cognitive reserve. Practical considerations include structured documentation of the level of frequency of language use, subjective linguistic competency, age of acquisition of languages, context of use, formal competency assessment of verbal fluency of languages, formal qualifications in each language and degree of language switching. These factors could be compiled in a structured linguistic competency questionnaire. We assert that by employing a more global and structured approach to linguistic competency we may be able to devise a rating scale which may provide an objective measure of linguistic proficiency.

## Discussion

### Bilingualism: A Global Public Health Strategy for Healthy Cognitive Aging

We now propose bilingualism as a significant public health initiative for healthy cognitive aging in LMICs and consider how this could be incorporated into policy. The G8 and WHO have highlighted that upscaling public health indicatives should be a focus on dementia management in LMICs (86). We encourage that adopting bilingualism into dementia policy in LMICs could be formalized through organizations such the Alzheimer's Disease International (87) and STRiDE: Strengthening responses to dementia in developing countries which advocate the public health approach (88).

There is an intrinsic value of delaying the onset of dementia (9). A delay of AD onset of 5 years may represent a 41% lower prevalence of lower cost of AD in 2050 (9). In HICs this delay may also equate to 2.7 additional life years and lower informal costs (9). We highlight that delaying the onset of dementia may be even more significant in LMICs where treatments are not freely available, and nursing and care needs are frequently placed on the children of those diagnosed with dementia. This may lead to significant losses of occupational and economic productivity amongst individuals of working age.

We discuss how bilingualism-based measures could be practically adopted within public health strategies in LMICs. One approach would be to promote bilingualism from childhood. Benson explored how bilingual language programs can be incorporated into school curriculum in LMICs using examples from Guinea-Bissau, Niger, Mozambique, and Bolivia (89). Benson suggests that bilingual teaching programs which are decentralized, linked to local culture and proficiency in mother tongue and include specialist language teachers may be more likely to be successful and welcomed by parents (89). Successful programs include the Nigerian six-year Yoruba medium project (90) and Guinea-Bissau bilingual project which integrated subject matter into themes, such as preventive health and improved gardening methods (91).

Whilst we have demonstrated the contribution of bilingualism toward cognitive reserve, we consider whether language learning in older age could be a feasible public health strategy to delay the onset of dementia in LMICs. Prior research has suggested that brain training may foster positive brain changes in healthy adults (92) and older people (93). This may indicate that mental stimulation may promote neuroplasticity even in the older adult. Learning a second language may cultivate healthy brain aging through engagement of additional brain networks (94).

A study which examined the benefits of one week of intensive Scottish Gaelic language training in older monolinguals revealed that these participants had improved in task switching cognitive tests (95). Improved cognitive performance was maintained at 9 months follow up in individuals who practiced Gaelic for at least 5 hours a week following the end of training (95). However, these findings were not replicated in a study of Spanish monolinguals who learnt Basque (96). Differences in the study design may have impacted the overall study findings. We suggest that future studies employ wide ranging and different bilingual linguistic profiles and are conducted in varied cultural and economic settings may help to discern more robust evidence in favor of bilingualism. Computer based approaches in language lessons has been explored (97), but we suggest less resource intense methods might be appropriate in LMICs.

Bak and colleagues (95) suggest that weekly 5 hours of minimum language training may be required to produce the cognitive benefits of bilingualism (95). In many LMICs where multiple languages are spoken, the principle language taught in schools may not necessarily be the mother tongue (89). Given this, we suggest that a personalized teaching program which incorporates local cultural practices and proficiency of inborne languages might be more beneficial in these settings. Conversely, in older populations where the proficiency may lie in the mother tongue, formal learning of a secondary language may be more advantageous in promoting healthy cognitive aging.

## Conclusion

This perspective has examined the role of bilingualism as a cognitive reserve factor from a wide range of evidential sources. We have explored studies of bilingualism conducted in both HICs and LMICs and reflected upon an important metanalysis that demonstrated that bilingualism is associated with a significant delay of onset of dementia. We determine that many key studies of bilingualism are limited by inconsistent working definitions of bilingualism and few have utilized objective measures of bilingual fluency. Furthermore, while several retrospective bilingualism studies have identified a significant delay in dementia onset this finding has not been replicated in prospective studies. We suggest that future research should explore the reasoning behind this discrepancy. Contextual challenges in LMICs including the high prevalence of illiteracy, HIV, socio-cultural and environmental disparities, and differing risk factors may complicate the overall picture.

Whilst finding a definitive treatment is the gold standard in dementia research, we suggest that public health measures that may promote the delay of clinical features of dementia, such as language lessons for the elderly or augmenting pre-existing bilingual proficiency in older age is important. This may be particularly salient in LMICs where cheap, pragmatic, and easily accessible approaches are warranted. If we are to harness the key benefits that bilingualism may provide, we encourage major stakeholders including governmental and health system providers to develop social programs and interventions to support the preservation of a second language.

## Data Availability Statement

The original contributions presented in the study are included in the article/supplementary material, further inquiries can be directed to the corresponding author/s.

## Author Contributions

SM was the principle author for this paper and derived the key topics for discussion in this paper. NT and VR provided general feedback and editorial comments. All authors contributed to the article and approved the submitted version.

## Conflict of Interest

The authors declare that the research was conducted in the absence of any commercial or financial relationships that could be construed as a potential conflict of interest.

## Footnotes

^1^A low and middle country has a GNI less than $12536 (5).
